# Applicability and validation of Franco, Willems and Willems II dental age estimation models in a population of Ceará, Brazil

**DOI:** 10.1038/s41598-025-34218-6

**Published:** 2026-01-06

**Authors:** Tácio Pinheiro Bezerra, Raíssa Araújo Gonçalves, Liz Magalhães Brito, Débora Duarte Moreira, Nikolaos Angelakopoulos, Marcelo Napimoga, Ademir Franco

**Affiliations:** 1 Forensic Dentistry, Ceara State Forensic Unit, Fortaleza, CE Brazil; 2 Division of Forensic Dentistry and Anatomy, Faculdade São Leopoldo Mandic, Campinas, SP Brazil; 3 Division of Oral Radiology, Faculdade São Leopoldo Mandic, Campinas, SP Brazil; 4 Department of Orthodontics and Dentofacial Orthopedics, University of Bern, Freiburgstrasse 7, 3010 Bern, Switzerland; 5 Division of Immunology, Faculdade São Leopoldo Mandic, Campinas, SP Brazil; 6 Department of Therapeutic Stomatology, Institute of Dentistry, Sechenov University, Moscow, Russia

**Keywords:** Age, Forensic dentistry, Teeth, Radiology, Health care, Medical research

## Abstract

Dental age assessment plays a crucial role in clinical and forensic contexts. For safer practices, however, the existing methods need to be tested. This study aimed to evaluate for the first time the applicability of Franco’s, Willems’, and Willems’ II dental age assessment models in a Northeastern Brazilian sample. The sample consisted of 500 panoramic radiographs (250 males, 250 females) of Brazilian individuals between 6 and 15.9 years from the State of Ceará. Chronological (CA) and estimated (EA) ages compared using mean error (ME), mean absolute error (MAE), and root mean square error (RMSE). Statistical comparisons were performed through generalized estimating equations (GEE). The mean CA of the sample was 11.0 years. The mean EA was 11.3 years for Franco’s model, 11.6 for Willems’, and 11.5 for Willems II. Franco’s model showed the smallest bias, with ME values being 0.25 and 0.21 years lower than Willems’ and Willems II, respectively (*p* < 0.001). Differences between CA and EA were minimal across sexes and not clinically relevant. Age-group analysis revealed similar performance up to 11.9 years. Overall, Franco’s model demonstrated better error metrics, but all the models showed comparable accuracy and consistency for dental age estimation in children and adolescents from Ceará, confirming their validity and suitability for both clinical and forensic applications in this population.

## Introduction

Dental age estimation is a procedure where the chronological age of a person is assessed through progressive or regressive odontological parameters^1^. Its application in practice extends from clinical to forensic science^2^. Clinically, dental age estimation can be applied in treatment planning and to help diagnose developmental conditions (especially in the fields of orthodontics, orofacial orthopedics, pediatric dentistry, and special care dentistry)^3^. From a forensic perspective, dental age estimation can contribute mainly to cases that involve asylum seekers and undocumented migrants, adoption, victims of pedo-pornography, legal liability, and human identification through the reconstructive approach of the biological profile of the deceased^4–10^.

Among the several methods available for dental age assessment, the seminal work published by Demirjian et al.^11^(1973) is one of the most popular. This radiographic system classifies of crown and root development of the permanent teeth in the third quadrant (except third molar) into eight stages, labeled from A to H^11^. Dental maturity scores are allocated for each tooth, summed and converted into age using sex-specific Table^11^ However, scientific studies testing Demirjian’s method in different populations demonstrated, however, consistent overestimations^12–16^. This limitation, combined with the method’s limited statistical approach, has led to its discouragement for practical applications aimed at dental age estimation, particularly within the forensic context^17^.

In 2001, Willems et al.^18^ revisited Demirjian’s staging technique and published a model trained with a sample of Belgian individuals. This model was tested in worldwide and validated based on performance^19,20^. For instance, in 2010, authors highlighted the Willems dental maturity scale as the best performing amongst four maturity scales and fifteen methods^21^. In 2021, a systematic review and meta-analysis confirmed the model as the best performing for application in Brazilian children^22^, and in 2025 an umbrella review corroborated the consistent minor differences between chronological and estimated ages observed with the model, underscoring its applicability for dental age estimation^23^. When compared with its precursor (Demirjian’s method), Willems’ model exhibited a noticeably reduced overestimation, namely in Brazilians, with Demirjian’s method yielding nearly twice the magnitude of overestimation observed with Willems^24^.

Following a similar methodological approach, a Brazilian-specific model (Franco: Forensic Radiological Analysis of Combined Orthopantomograms) was developed applying Demirjian’s technique and combining 5,017 panoramic radiographs from three geographic regions of the country (Southeastern, Central-Western and South)^25^. This approach enabled the reduction of mean absolute errors between chronological and estimated ages compared to Willems’ model, providing additional reference values for dental age assessment of Brazilian children and adolescents^25^. Given the recent development of this model, it has not yet been assessed across different regions of the country nor benchmarked against other dental age estimation methods.

Based on the exposed scientific justification, the present study aimed to (I) apply and test the Franco model for dental age assessment in children and adolescents from Northeastern Brazil, and (II) compare it with Willems and Willems II Belgian models’ performance.

## Materials and methods

### Study design and ethical aspects

This study was designed as an observational-analytical, cross-sectional study, with retrospective sample collection and quantitative data assessment. Reporting followed the Strengthening the Reporting of Observational Studies in Epidemiology (STROBE) checklist, part of Enhancing the Quality and Transparency of Health Research (EQUATOR) network (https://www.equator-network.org/*).* This retrospective study was approved by the Institutional board (Ethics Committee for Human Research protocol number: 92882425.2.0000.5374). Signed informed consent was obtained from the legal guardian(s) of all participants whose radiographs were included in the study.Institutional ethical approval was obtained following the Declaration of Helsinki (2024).

### Settings and participants

The sample was obtained from a private oral radiology service located in the State of Ceará, Northeastern Brazil, and it consisted of panoramic radiographs previously acquired for dental treatment purposes. The radiographs available in the digital database had been produced between 2015 and 2025 and were assessed retrospectively, meaning that no patient was prospectively exposed to ionizing radiation for research purposes. The inclusion criteria comprised panoramic radiographs of Brazilian male and female from the State of Ceará, with complete records of sex, date of birth, and date of image acquisition. The exclusion criteria, on the other hand, included radiographs of individuals younger than 6 years or older than 15.99 years, bilateral absence of permanent mandibular teeth, image artifacts hampering visualization, teeth presenting extensive carious lesions, large restorations, or root canal treatments; and cases showing visible anomalies, bone deformities, or cystic/tumoral lesions affecting the mandibular region. The universe of potentially available radiographs within the medical imaging database comprised 13,331 radiographs. Adopting the respective age range frame and sample size by Gelbrich et al.^26^ (2020), and also considering the calculation for dental age estimation via panoramic radiographs in the Northeastern Brazilian population (same region addressed in the present study set with 95% confidence interval, 5% type I error, and 80% statistical power) presented by Nóbrega et al.^27^ (2025), our sample size was established as 500 images. Following the recommendations of the Study Group on Forensic Age Diagnostics (SGFAD) of the German Association of Forensic Medicine an even age distribution was accomplished to balance males (*n* = 250) and females (*n* = 250) and similarly allocate them in age categories (Table 1).

**Table 1:** Sample distribution based on age and sex.

	Sex		Total	*p*
	Females	Males		
Total	250 (50.0%)	250 (50.0%)	500 (100.0%)	
**Age categories**				1.000
6–6.9.9	25 (10.0%)	25 (10.0%)	50 (10.0%)	
7–7.9.9	25 (10.0%)	25 (10.0%)	50 (10.0%)	
8–8.9.9	25 (10.0%)	24 (9.6%)	49 (9.8%)	
9–9.9.9	24 (9.6%)	26 (10.4%)	50 (10.0%)	
10–10.9.9	26 (10.4%)	25 (10.0%)	51 (10.2%)	
11–11.9.9	25 (10.0%)	25 (10.0%)	50 (10.0%)	
12–12.9.9	24 (9.6%)	25 (10.0%)	49 (9.8%)	
13–13.9.9	26 (10.4%)	24 (9.6%)	50 (10.0%)	
14–14.9.9	25 (10.0%)	26 (10.4%)	51 (10.2%)	
15–15.9.9	25 (10.0%)	25 (10.0%)	50 (10.0%)	

Age expressed in years; *p* value representing chi-square test showing lack of statistically significant differences (considering statistical significance at 5%) between the number of individuals across age categories.

### Variables and data sources

**Fig. 1:**
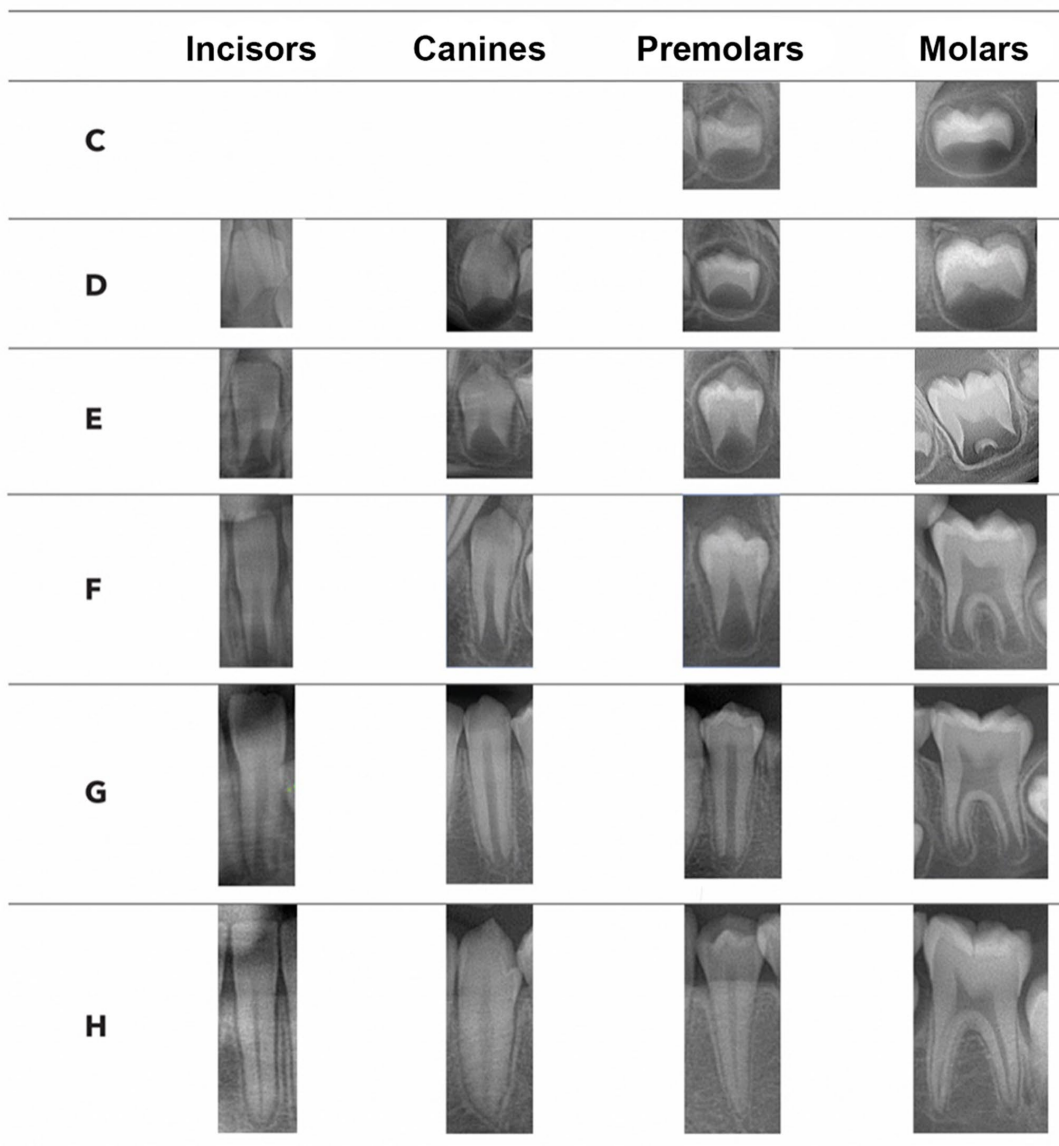
Radiographic representation of Demirjian’s^1^ staging system for incisors, canines, premolars and molars, considering the stage coverage enabled by the present sample.

The study variables were the individuals’ sex, chronological age (CA) and estimated age (EA). CA was calculated as the difference between date of image acquisition and date of birth and was converted into a categorical variable. The age categories were from 6 to 6.99 years to 15–15.99.99 years. The EA was calculated after the application of three dental age assessment models: Franco (Franco et al. 2024)^25^, Willems (Willems et al., 2001)^18^ and Willems II (Willems et al., 2010)^24^. All the models were based on Demirjian’s staging technique^11^. This system classifies crown and root formation of permanent incisors, canines, premolars and molars, into eight stages from the initial signs of cusp mineralization (A) to apical closure (H) (Fig. 1). The system considers the mandibular teeth of the left side and does not include third molars. For Franco’s and Willems’ models, sex-specific maturity scores were used, while Willems II single non-specific sex table was applied to both sexes. Image analysis was conducted by the main observer over a period of 6 months, randomly across masked files to enable a blind staging (images previously preprocessed by cropping out patients’ sex and age) and not exceeding 25 radiographs per day, to avoid visual fatigue. The radiographs were assessed in a personal computer with a 16-inch Dell display (Dell Inc., Round Rock, Texas, USA) equipped with an Intel ore™ i7 and Adobe Photoshop CS5 (Adobe Systems Inc., San Jose, California, USA) for image visualization. In this software, the digital radiographs were assessed, and teeth were staged allowing adjustments of brightness and contrast and up to 200% of image magnification.

### Operator-dependent bias

The operator-dependent bias was evaluated through intra- and inter-observer reliability tests. For this purpose, 10% of the sample (*n* = 50) radiographs were reassessed by the main observer 30 days after the initial analysis, and an equivalent subset was independently evaluated by a second trained observer – both were calibrated^25^ forensic odontologists with three and thirteen years of experience, respectively. Comparisons between the two assessments performed by the main observer were used to determine intra-observer reliability, while comparisons between the evaluations of both observers were used to assess inter-observer agreement. The level of observer reliability was quantified using weighted Kappa statistics. In this context, the stages between observers were compared and not the EA.

### Statistical analysis

The difference between CA and EA was assessed through error estimates, namely mean error (ME), mean absolute error (MAE), and root mean square error (RMSE) according to sex (female and male) and age categories (i.e.: 6–6.9.9; 7–7.9; 8–8.9; 9–9.9; 10–10.9; 11–11.9; 12–12.9; 13–13.9; 14–14.9; and 15–15.9). Bland-Altman plots were also used to visualize comparisons. The number of participants by age group and sex was compared using chi-square tests.

Comparisons of ME between Franco’s, Willems’ and Willems’ II models were performed using regressions based on generalized estimating equations (GEE) with a Gaussian family and identity link function. For comparisons of MAE and RMSE, GEE regressions were also applied, but using a gamma family and log link function, as both estimates showed right-skewed distributions. Thus, the regression coefficients for ME represented mean differences (in absolute terms), whereas the coefficients for MAE and RMSE represented ratios (in relative terms). All analyses were performed using Stata software, version 18 (StataCorp LLC, College Station, TX, USA), considering a significance level of 5%.

## Results

The intra-observer agreement was above 0.82, while the inter-observer agreement was above 0.77, considering all the seven teeth.

The mean CA of the sample was 11 years. The mean EA was 11.3 years using Franco’s models, 11.6 years using Willems’ models and 11.5 years using Willems’ II model. It was observed that, for the younger age groups, Franco’s model tended to yield higher age estimates compared to the other models, whereas for the older age groups, it produced lower estimates (Table 2).

**Table 2:** Mean chronological and estimated ages for each dental age assessment models.

	CA (SD)	EA (SD)		
		Franco	Willems	Willems II
**Overall mean**	11.0 (2.9)	11.3 (2.8)	11.6 (3.2)	11.5 (3.2)
**Age categories**				
6-6.9	6.5 (0.3)	7.1 (0.6)	6.9 (0.7)	6.9 (0.7)
7-7.9	7.5 (0.3)	8.1 (0.7)	8.0 (0.7)	8.0 (0.7)
8-8.9	8.5 (0.3)	9.0 (1.0)	8.9 (1.0)	8.9 (1.1)
9-9.9	9.4 (0.3)	9.7 (0.8)	9.7 (0.9)	9.6 (0.8)
10-10.9	10.5 (0.3)	10.9 (1.1)	11.0 (1.2)	10.9 (1.2)
11-11.9	11.5 (0.3)	12.3 (1.3)	12.4 (1.5)	12.3 (1.6)
12-12.9	12.5 (0.3)	13.1 (1.2)	13.3 (1.5)	13.3 (1.6)
13-13.9	13.5 (0.3)	14.0 (0.9)	14.6 (1.3)	14.6 (1.4)
14-14.9	14.4 (0.3)	14.4 (0.7)	15.1 (1.1)	15.1 (1.2)
15-15.9	15.5 (0.3)	14.7 (0.3)	15.7 (0.6)	15.7 (0.6)
**Sex**				
Females	11.0 (2.9)	11.4 (2.7)	11.6 (3.2)	11.8 (3.1)
Males	11.0 (2.9)	11.3 (2.8)	11.5 (3.2)	11.3 (3.2)

Age expressed in years; CA: chronological age; EA: estimated age; SD: standard deviation.

The error estimates comparing CA and EA for the analytical sample are presented in Table 3. Franco’s model produced the closest estimates (smallest ME). When comparing the ME among models, statistically significant differences were found (*p* < 0.001). The ME with Franco’s model was 0.25 and 0.21 years lower than those of the Willems’ and Willems’ II models, respectively. Regarding MAE and RMSE, the estimated differences were multiplicative: the absolute errors for Willems’ model were 15% higher than those of Franco’s. As for the RMSE, Willems’ model showed 43% higher errors and Willems’ II 45% higher errors compared to Franco’s, all with statistical significance (*p* < 0.001) (Table 3).

**Table 3:** Error metrics after comparing chronological and estimated ages for each model, and comparison of between the errors of the willems’ and willems’ II models with those from Franco’s.

Metrics	Franco	Willems	Willems II	Franco vs. Willems	Franco vs. Willems II
ME^a^	−0.33 (−0.42; −0.24)	−0.58 (−0.68; −0.49)	−0.54 (−0.64; −0.44)	−0.25 (−0.29; −0.21)*	−0.21 (−0.25; −0.16)*
MAE^b^	0.83 (0.77; 0.88)	0.95 (0.88; 1.02)	0.95 (0.88; 1.02)	1.15 (1.10; 1.19)*	1.15 (1.11; 1.20)*
RMSE^b^	1.04 (0.97; 1.11)	1.25 (1.15; 1.33)	1.25 (1.16; 1.34)	1.43 (1.34; 1.53)*	1.45 (1.36; 1.55)*

^a^Comparison between methods performed using regression based on generalized estimating equations with a Gaussian family and identity link function; ^b^Comparison between methods performed using regression based on generalized estimating equations with a gamma family and log link function; *: *p* < 0.001. ME: mean error; MAE: mean absolute error; RMSE: root mean squared error.

The comparison by sex showed similar findings to those observed for the total sample, with Franco’s model producing significantly smaller errors than the Willems’ model among females. For males, the comparison between Franco’s and Willems’ models also revealed smaller errors for the former (*p* < 0.001). However, when comparing Franco’s and Willems’ II, no significant difference was found for the ME, although Willems II presented 7% higher absolute errors (*p* < 0.05) and 30% higher RMSE (*p* < 0.001) (Table 4).

**Table 4:** Error metrics after comparing chronological and estimated ages for each model, and comparison of the errors from willems’ and willems’ II models with those from franco’s according to sex.

Sex	Franco	Willems	Willems II	Franco vs. Willems	Franco vs. Willems II
**Females**					
ME^a^	−0.35 (−0.48; −0.22)	−0.55 (−0.69; −0.41)	−0.78 (−0.92; −0.65)	−0.20 (−0.26; −0.14)*	−0.43 (−0.49; −0.37)*
MAE^b^	0.86 (0.78; 0.94)	0.96 (0.86; 1.06)	1.05 (0.95; 1.16)	1.12 (1.06; 1.19)*	1.23 (1.16; 1.30)*
RMSE^b^	1.08 (0.98; 1.17)	1.26 (1.13; 1.38)	1.35 (1.22; 1.47)	1.37 (1.25; 1.50)*	1.57 (1.43; 1.73)*
**Males**					
ME^a^	−0.31 (−0.43; −0.19)	−0.62 (−0.75; −0.48)	−0.29 (−0.43; −0.15)	−0.31 (−0.35; −0.26)*	0.02 (−0.03; 0.07)^¥^
MAE^b^	0.80 (0.72; 0.87)	0.93 (0.83; 1.03)	0.85 (0.76; 0.95)	1.17 (1.11; 1.24)*	1.07 (1.01; 1.13)^§^
RMSE^b^	1.01 (0.89; 1.11)	1.23 (1.09; 1.36)	1.15 (1.01; 1.27)	1.51 (1.38; 1.65)*	1.30 (1.19; 1.43)*

^a^Comparison between methods performed using regression based on generalized estimating equations with a Gaussian family and identity link function; ^b^Comparison between methods performed using regression based on generalized estimating equations with a gamma family and log link function; *: *p* < 0.001; ^§^: *p* < 0.05; ^¥^: *p* ≥ 0.05; ME: mean error; MAE: mean absolute error; RMSE: root mean squared error.

Age group comparisons indicated no statistically significant differences among the models for the youngest age categories (6 to 10.9 years). From 11 to 11.9 years onward, all error estimates from Franco’s model were significantly smaller than those from Willems’ and Willems’ II models (Table 5).

**Table 5:** Error metrics after comparing chronological and estimated ages for each model, and comparison of the errors from the willems’ and willems’ II models with those from the franco’s according to age categories.

Category	Franco	Willems	Willems II	Franco vs. Willems	Franco vs. Willems II
**6–6.9.9**					
ME^a^	−0.56 (−0.69; −0.43)	−0.34 (−0.52; −0.16)	−0.39 (−0.57; −0.22)	0.22 (0.14; 0.30)*	0.17 (0.09; 0.25)*
MAE^b^	0.60 (0.48; 0.71)	0.56 (0.44; 0.69)	0.56 (0.43; 0.69)	0.94 (0.83; 1.08)^¥^	0.95 (0.83; 1.08)^¥^
RMSE^b^	0.72 (0.59; 0.84)	0.71 (0.57; 0.83)	0.72 (0.57; 0.85)	0.96 (0.79; 1.17)^¥^	1.00 (0.82; 1.22)^¥^
**7–7.9.9**					
ME^a^	−0.58 (−0.80; −0.37)	−0.52 (−0.72; −0.31)	−0.49 (−0.71; −0.28)	0.07 (−0.01; 0.14)^¥^	0.09 (0.02; 0.17)^§^
MAE^b^	0.75 (0.58; 0.92)	0.69 (0.52; 0.85)	0.71 (0.55; 0.86)	0.92 (0.83; 1.02)^¥^	0.95 (0.85; 1.05)^¥^
RMSE^b^	0.95 (0.72; 1.14)	0.89 (0.67; 1.06)	0.90 (0.65; 1.09)	0.86 (0.72; 1.03)^¥^	0.88 (0.73; 1.05)^¥^
**8–8.9.9**					
ME^a^	−0.49 (−0.75; −0.22)	−0.42 (−0.69; −0.15)	−0.39 (−0.68; −0.11)	0.06 (−0.02; 0.15)^¥^	0.09 (0.01; 0.17)^§^
MAE^b^	0.78 (0.58; 0.98)	0.73 (0.53; 0.94)	0.78 (0.57; 0.99)	0.94 (0.85; 1.05)^¥^	1.00 (0.91; 1.11)^¥^
RMSE^b^	1.04 (0.64; 1.32)	1.02 (0.58; 1.32)	1.06 (0.67; 1.33)	0.96 (0.84; 1.11)^¥^	1.03 (0.90; 1.19)^¥^
**9–9.9.9**					
ME^a^	−0.28 (−0.50; −0.06)	−0.26 (−0.51; 0.00)	−0.20 (−0.44; 0.03)	0.03 (−0.06; 0.12)^¥^	0.08 (−0.01; 0.17)^¥^
MAE^b^	0.70 (0.57; 0.82)	0.72 (0.55; 0.89)	0.71 (0.57; 0.84)	1.03 (0.91; 1.16)^¥^	1.01 (0.90; 1.14)^¥^
RMSE^b^	0.82 (0.68; 0.95)	0.93 (0.73; 1.09)	0.85 (0.70; 0.97)	1.27 (1.06; 1.52)^§^	1.06 (0.88; 1.27)^¥^
**10–10.9.9**					
ME^a^	−0.44 (−0.76; −0.13)	−0.49 (−0.81; −0.16)	−0.39 (−0.73; −0.05)	−0.04 (−0.12; 0.04)^¥^	0.05 (−0.03; 0.14)^¥^
MAE^b^	0.91 (0.69; 1.13)	0.96 (0.75; 1.18)	0.97 (0.74; 1.20)	1.06 (0.97; 1.16)^¥^	1.07 (0.97; 1.16)^¥^
RMSE^b^	1.19 (0.92; 1.40)	1.23 (0.97; 1.45)	1.27 (0.97; 1.50)	1.08 (0.96; 1.22)^¥^	1.14 (1.01; 1.29)^§^
**11–11.9.9**					
ME^a^	−0.74 (−1.11; −0.36)	−0.87 (−1.29; −0.44)	−0.77 (−1.21; −0.32)	−0.13 (−0.24; −0.03)^§^	−0.03 (−0.13; 0.07)^¥^
MAE^b^	1.21 (0.95; 1.46)	1.33 (1.03; 1.64)	1.37 (1.06; 1.67)	1.11 (1.03; 1.19)^¥^	1.13 (1.06; 1.22)*
RMSE^b^	1.49 (1.20; 1.74)	1.71 (1.29; 2.05)	1.73 (1.31; 2.07)	1.31 (1.15; 1.50)*	1.34 (1.18; 1.54)*
**12–12.9.9**					
ME^a^	−0.59 (−0.95; −0.24)	−0.89 (−1.32; −0.45)	−0.80 (−1.25; −0.35)	−0.29 (−0.40; −0.18)*	−0.20 (−0.31; −0.09)*
MAE^b^	1.18 (1.00; 1.37)	1.45 (1.16; 1.73)	1.40 (1.10; 1.70)	1.22 (1.14; 1.31)*	1.18 (1.10; 1.27)*
RMSE^b^	1.35 (1.12; 1.54)	1.74 (1.38; 2.04)	1.74 (1.37; 2.04)	1.67 (1.49; 1.88)*	1.66 (1.48; 1.87)*
**13–13.9.9**					
ME^a^	−0.50 (−0.72; −0.27)	−1.12 (−1.46; −0.77)	−1.07 (−1.43; −0.71)	−0.62 (−0.75; −0.49)*	−0.57 (−0.70; −0.44)*
MAE^b^	0.76 (0.61; 0.91)	1.32 (1.05; 1.60)	1.32 (1.04; 1.60)	1.74 (1.60; 1.89)*	1.73 (1.59; 1.88)*
RMSE^b^	0.93 (0.76; 1.07)	1.64 (1.38; 1.87)	1.65 (1.39; 1.87)	3.14 (2.76; 3.57)*	3.15 (2.77; 3.58)*
**14–14.9.9**					
ME^a^	0.04 (−0.16; 0.24)	−0.74 (−1.06; −0.42)	−0.69 (−1.02; −0.36)	−0.78 (−0.90; −0.66)*	−0.73 (−0.85; −0.61)*
MAE^b^	0.58 (0.46; 0.70)	1.22 (1.06; 1.37)	1.24 (1.09; 1.39)	2.09 (1.78; 2.46)*	2.13 (1.81; 2.51)*
RMSE^b^	0.72 (0.57; 0.84)	1.34 (1.20; 1.46)	1.35 (1.23; 1.46)	3.47 (2.60; 4.65)*	3.55 (2.65; 4.75)*
**15–15.9.9**					
ME^a^	0.81 (0.69; 0.93)	−0.20 (−0.37; −0.04)	−0.19 (−0.36; −0.02)	−1.02 (−1.10; −0.94)*	−1.00 (−1.08; −0.92)*
MAE^b^	0.81 (0.69; 0.93)	0.48 (0.37; 0.59)	0.48 (0.36; 0.59)	0.59 (0.47; 0.74)*	0.58 (0.47; 0.73)*
RMSE^b^	0.91 (0.78; 1.03)	0.62 (0.45; 0.75)	0.62 (0.46; 0.75)	0.46 (0.33; 0.63)*	0.47 (0.34; 0.65)*

^a^Comparison between methods performed using regression based on generalized estimating equations with a Gaussian family and identity link function; ^b^Comparison between methods performed using regression based on generalized estimating equations with a gamma family and log link function; *: *p* < 0.001; ^§^: *p* < 0.05; ^¥^: *p* ≥ 0.05; ME: mean error; MAE: mean absolute error; RMSE: root mean squared error.

**Fig. 2:**
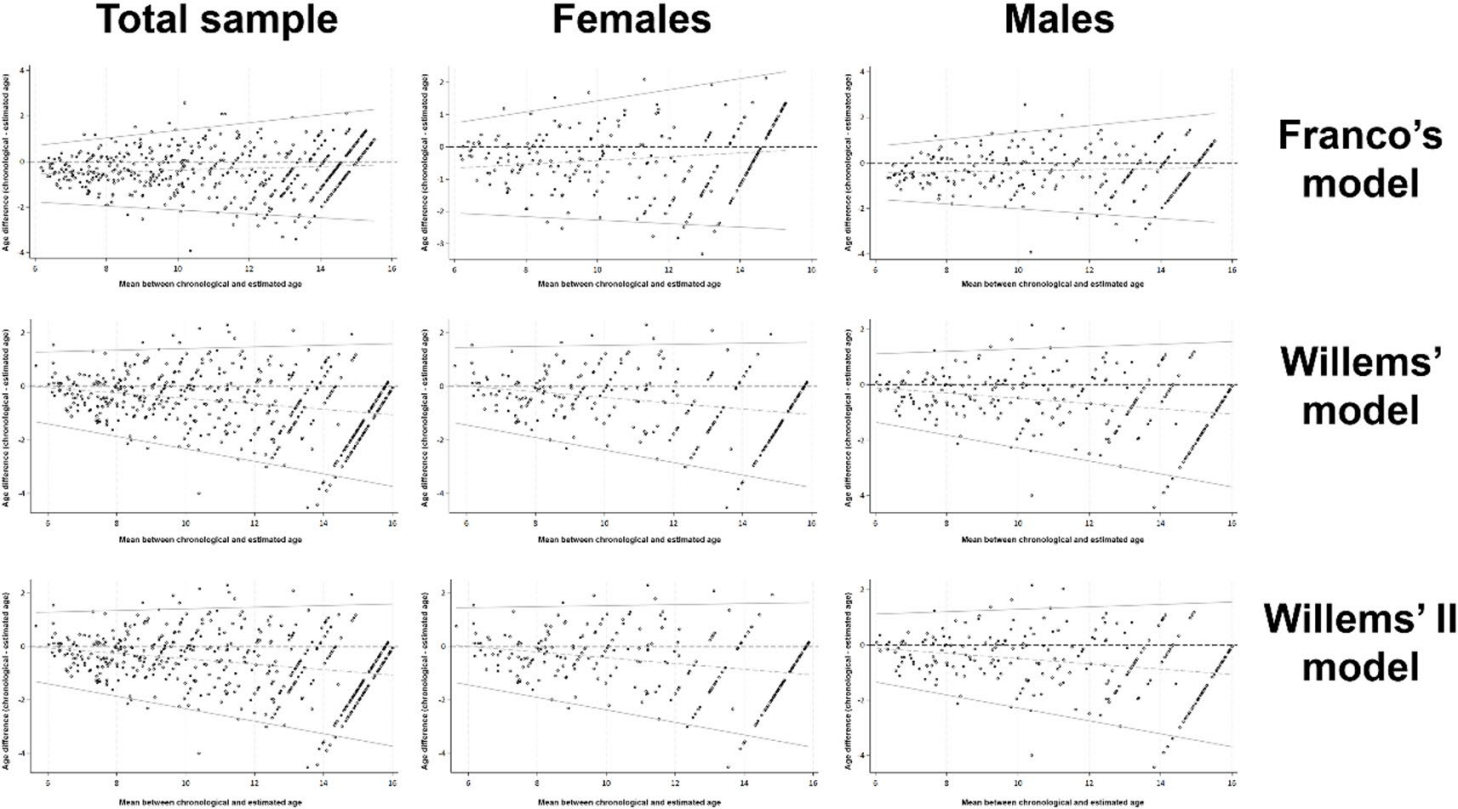
Bland-Altman plots displaying the differences between chronological and estimates for the total sample, and for females and males, considering each dental age assessment model, namely Franco’s, Willems’ and Willems’ II.

For all three models, the Bland-Altman plots showed wider limits of agreement in older age groups. However, the differences between CA and EA for Franco’s model were smaller compared to the other two models. The agreement analyses stratified by sex showed greater dispersion compared to the total sample, yet the differences between CA and EA with Franco’s model remained consistently below the other models (Fig. 2).

## Discussion

Dental age estimation studies can be categorized at least into three types according to their target population. Studies designed to address the I) adult population often revisit regressive morphological changes of the teeth derived from Gustafson’s parameters^28–30^. The scientific literature focused on II.1) subadults can address third molar formation^31–33^, with especial applicability normally between the ages of 16 and 21 years when the assessment of legal majority is necessary^34,35^; and II.2) deciduous/permanent tooth formation when practical applicability extends to clinical (e.g. orthopedic and orthodontic treatment planning, and investigation of systemic conditions)^36,37^ and legal/forensic (e.g. victims of violence and exploitation, adoption, and reconstructive human identification) purposes^38–40^. The present study focused on a subadult population of children and adolescents up to 15.9 years of age. In this context, three pioneering aspects should be highlighted: it was the first application of the Franco dental age assessment model to a Northeastern Brazilian population from the State of Ceará; the first evaluation of the Willems II model among Brazilian individuals; and the first direct comparison of both methods with the original Willems model.

To address the innovative aspects of the present study and its contribution to the existing body of scientific knowledge, it is essential to recall what is already known. The Willems model has been extensively applied to the Brazilian population, largely due to the country’s continental dimensions and population of approximately 210 million inhabitants. This demographic and geographic diversity has driven the scientific and investigative interest of researchers in testing the model across different regions. Brazil is divided into five macro-regions: Southern, Southeastern, Central-Western, Northern, and Northeastern. The first three regions have already been represented in studies employing the Willems dental age assessment model^25,36,41,42^, and the inherent outcomes have confirmed the model’s applicability. For instance, within 1,990 children and adolescents up to 15.9 years, sampled from Rio de Janeiro, Willems’ model showed differences between CA and EA that were not superior to 0.38 years^36^. Samples from the Northern and Northeastern regions, however, remained underinvestigated. The present study therefore contributed by filling this geographic and scientific gap, expanding the understanding and applicability of dental age estimation within the Brazilian context.

The performance of Willems’ model in the present Northeastern Brazilian sample demonstrated an overall mean difference between CA and EA about 0.6 years, with minimal differences based on sex. Slight increase in error metrics were observed from 6 to 11.9 years, peaking in the age category between 12 and 12.9 years. A previous study also in the State of Ceará, Brazil^43^, have applied Demirjian’ dental age assessment approach and observed pronounced differences between CA and EA. More specifically, the authors observed differences that were higher than one year in 68% and 72% of the studied age categories between 7 and 13 years, for males and females, respectively^43^. With their findings, the authors justified the development of population-specific maturity scores as an alternative for tailored dental age assessment^43^. Internationally, systematic reviews and meta-analyses^19,20^ have endorsed Willems’ model for dental age estimation demonstrating that this approach leads to considerably lesser overestimations^20^ compared to other existing methods for children and young adolescents. More specifically, the model has demonstrated negligible overall pooled overestimations, without showing statistically significant differences between the mean age of different geographic regions^19^. The present study confirms Willems’ model applicability for children and adolescents, adding a valuable tool to the armamentarium of clinical and forensic dental services in the State of Ceará, Brazil.

Willems’ II model showed overall differences between CA and EA that were similar to Willems’ model, but higher discrepancies were observed between males and females. Compared to the overall CA, the EA with Willems’ II showed a difference of 0.3 years for males and 0.8 years for females, while Willems’ original model showed differences of 0.5 and 0.6 years, respectively. This may be explained by the fact that Willems’ II is a non-sex-specific model. Nonetheless, this model was applied not only to the combined sample, but also separately for males and females in the present study specifically to evaluate how age estimation would perform within each group. Despite the slight differences observed between sexes, Willems II consistently performed comparably to the original Willems model, confirming its applicability and validity for dental age assessment in the studied population. A previous study with a South American sample from Venezuela compared Willems’ models, concluding that both were comparably applicable to the studied sample^44^. It must be noted that Willems’ II represents an important resource for forensic purposes that aim to identify young individuals, especially in skeletal remains. This is because according to the Scientific Working Group for Forensic Anthropology (SWGANTH) sex estimation is unadvised for immature individuals, mainly below the age of 12 years^45^ (given the lack of hormonal differentiation). In this case, a non-sex-specific tool becomes helpful to support dental and anthropological examinations as a viable option for age assessment.

Franco’s model presented the lowest overall mean difference between CA and EA, with ME showing an overestimation of −0.33 years, decreasing the MAE by 13% compared to Willems’ and Willems’ II models. Negligible differences of −0.04 (ME) and 0.06 (MAE) were observed based on sex, suggesting similar the model’s performance for males and females. All the error metrics showed statistically significant differences compared to the error metrics of Willems’ and Willems’ II (except for the ME) models. However, the differences were between 0.02 and − 0.43 years, not exceeding three months of age and being more statistically than clinically significant. When performed based on age category, the comparisons showed statistically significant differences that were predominant after 12 years. In the age bracket 12–14.9.9, Franco’s model demonstrated better error metrics, with ME differences exceeding six months of age (expressed as overestimations) between 13 and 14.9 years.

A twofold explanation could be considered to justify the positive performance of Franco’s model in the sampled population of Ceará, Brazil. Firstly, the nature of Franco’s model that considered a large sample of geographically combined panoramic radiographs from three Brazilian regions^25^. Secondly, it must be noted that Franco’s model prioritize the staging-scoring of permanent second molars, attributing higher scores to these teeth^25^. This methodological decision was originally accomplished because the second molar region was considered of easier visualization compared to other anatomic regions of the mandible. Visualization of the anterior region of the mandible and teeth can be affected by the superimposition of radiopaque and radiolucent images from the vertebrae and intervertebral spaces^46^. Moreover, this region can be more affected by horizontal magnification and other distortions inherent to several image acquisition variables, especially patient positional settings^47,48^. Interestingly, in 2025, authors have invested which tooth could lead to the best age prediction using the 3rd version of the London Atlas application and concluded that despite different teeth could better predict in different age categories, second molars presented lower mean differences and mean absolute differences between CA and EA^49^. This is a positive aspect confirming the importance of second molars for dental age estimation up to the age of 16 years.

While the present study expands existing literature by adding validated dental age estimation tools for the studied population and contributing to the body of knowledge, it must be noted that it is not without limitations. The panoramic radiographs were obtained from a single radiology center in the Northeastern region of Brazil, which may limit the representativeness of the findings. More studies are necessary to include samples from other centers and unexplored territories enabling external validations, preferably by independent research teams. The latter being important for achieving a more comprehensive interpretation of the model’s performance and generalizability between experts. The additional studies should also cover cross-border populations, especially in South America, where Brazil is contiguous with 10 out of 12 other countries in the continent, meaning that reference studies are necessary to support dental age estimation in case of clandestine immigration, criminal liability involving the foreign^50^ and multinational disaster victim identification.

## Conclusion

Franco’s, Willems’ and Willems’ II dental age assessment model have been tested and validated for application in children and adolescents from Ceará, Brazil. All the models demonstrated proper applicability that can be useful for clinical and forensic purposes. Overall, Franco’s model showed better error metrics compared to the other models, demonstrating differences that can be statistically significant but not necessarily of clinical relevance. Differences based on sex did not exceed six months, and comparison across age categories showed more similar performances between the models up to the age of 11.9 years. In practice, dental age estimation in individuals from the Northeastern State of Ceará, Brazil, can be examined by means of Franco’s Brazilian dental age assessment model, Willems’ sex-specific Belgian model, and Willems’ II non-sex-specific Belgian model.

## Data Availability

The data supporting this study’s findings are available from the project supervisor, Prof. Ademir Franco, upon reasonable request and with institutional permission.
